# Attack and Success of Native and Exotic Parasitoids on Eggs of *Halyomorpha halys* in Three Maryland Habitats

**DOI:** 10.1371/journal.pone.0150275

**Published:** 2016-03-16

**Authors:** Megan V. Herlihy, Elijah J. Talamas, Donald C. Weber

**Affiliations:** 1 Invasive Insect Biocontrol and Behavior Laboratory, USDA Agricultural Research Service, Beltsville, Maryland, United States of America; 2 Systematic Entomology Laboratory, USDA Agricultural Research Service, and Smithsonian Institution, Washington, DC, United States of America; University of Basilicata, ITALY

## Abstract

Egg parasitoids of the exotic invasive brown marmorated stink bug, *Halyomorpha halys* (Stål), were investigated using lab-reared fresh (live) and frozen (killed) lab-reared sentinel egg masses deployed for 72h on foliage in three habitats—woods, orchard, and soybean field—in Maryland, USA, in summer 2014. Four native hymenopteran species, *Telenomus podisi* Ashmead (Scelionidae), *Trissolcus euschisti* (Ashmead) and *Tr*. *brochymenae* Ashmead (Scelionidae), and *Anastatus reduvii* (Howard) (Eupelmidae), developed and emerged from *H*. *halys* eggs. One exotic parasitoid, *Trissolcus japonicus* (Ashmead), emerged, providing the first known occurrence of this species in North America. Native parasitoids emerged from frozen eggs significantly more often than from fresh eggs (89.3% of egg masses and 98.1% of individual eggs), whereas the exotic *Tr*. *japonicus* did not show a similar difference, strongly suggesting adaptation to *H*. *halys* as a host by *Tr*. *japonicus* but not by the native species. Parasitoids were habitat-specific: all three *Trissolcus* species were significantly more likely to occur in the woods habitat, whereas *Te*. *podisi* was found exclusively in the soybean field. Further investigations are required to elucidate evolving host-parasitoid relationships, habitat specificity, and non-target effects of *Tr*. *japonicus* over the expanded range of *H*. *halys* in North America.

## Introduction

The brown marmorated stink bug, *Halyomorpha halys* (Hemiptera: Pentatomidae), is native to Asia and was first discovered in the United States in 2001 in Allentown, Pennsylvania [1]. This polyphagous pest has since spread to 41 states causing economic damage to a wide variety of both fruit and vegetable crops and posing a nuisance in the fall and winter as the adults overwinter in residential and commercial buildings. The range of *H*. *halys* is expanding in Europe and North America, and is a threat to crops world-wide [2–6]. Chemical pesticides have met with mixed success in controlling *H*. *halys*, and deployment of pheromone baited traps has so far served to monitor widely their numbers and spread [7,8], but not to manage populations.

A potential long-term and low-cost strategy for control of *H*. *halys* in North American environments is biological control using egg parasitoid wasps. Although several egg parasitoid species in the genus *Trissolcus* (Hymenoptera: Scelionidae) attack and successfully parasitize *H*. *halys* in Asia, currently egg parasitoids native to the United States parasitize *H*. *halys* with very limited success [9]. Instead, native parasitoids typically fail to develop and/or emerge from *H*. *halys* eggs which thereby act as a sink for native parasitoids in both North America [10] and Europe [11]. Beginning in 2007, the USDA-ARS Beneficial Insect Introduction Research Unit (ARS/BIIRU) brought species of *Trissolcus* from China, Korea and Japan to quarantine laboratories in the U.S. for evaluation as potential biological control agents of *H*. *halys* in North America. *Trissolcus japonicus* (Ashmead) and *Trissolcus cultratus* (Mayr) predominated in the Asian collections obtained from *H*. *halys* [12]. Prior to possible implementation of classical biological control (the introduction of exotic natural enemies to control exotic pest species), several researchers had begun investigating the ability of native parasitoids to successfully parasitize *H*. *halys* eggs by placing sentinel egg masses in the field (e.g., [9,10]). Unexpectedly, we found that the exotic *Tr*. *japonicus* was present in the United States and attacking *H*. *halys* eggs in the field, presumably as a result of an accidental introduction [13].

Sentinel egg masses of exotic hosts such as *H*. *halys* may be subject to low rates of successful parasitism not only because native parasitoids may be maladapted to exotic hosts, but also because of a lack of host finding cues available to female parasitoids. Parasitoids use host insect and plant volatiles as long-range host finding cues [14]. These cues are not available to the female parasitoids responding to sentinel egg masses laid by colony insects and then placed out into the habitat. Jones et al. [9] showed that naturally laid *H*. *halys* egg masses in orchards experienced higher rates of parasitism than sentinel egg masses placed in the same habitat, likely due to the increase in host finding cues available to the wasps.

The primary goal of our study was to quantify field rates of attack and successful parasitism of *H*. *halys* eggs, using frozen eggs [11] as described below, fresh eggs from a lab colony, and eggs naturally laid by field-caged *H*. *halys* females. Previous studies in the U.S. have deployed sentinel egg masses exclusively in managed crops such as orchards, vineyards, tree nursery and row crops [5,6]. We decided to include a wooded unmanaged habitat, along with two agroecosystems, to expand our knowledge of field parasitism of *H*. *halys*.

We tested the following hypotheses: (1) Frozen sentinel egg masses yield higher rates of successful parasitism than fresh sentinel or cage laid egg masses at all sites; (2) Egg masses laid inside of mesh cages by *H*. *halys* in the field should result in higher parasitism than comparable colony laid sentinel egg masses; (3) Parasitoid species are habitat specific; and (4) Native parasitoids only successfully parasitize, develop, and emerge from sentinel eggs that were dead prior to deployment. In addition, upon discovery of the exotic parasitoid *Trissolcus japonicus*, we further hypothesized that its successful emergence from its Asian host *H*. *halys* would exceed that of North American native parasitoids.

## Materials and Methods

Field permits were not required because all research was conducted at sites on land within our USDA ARS facility in Beltsville, MD. Our studies did not involve endangered or protected species.

We conducted a sentinel egg mass study to assess parasitoids of the invasive pest, *H*. *halys*, in three field habitats during the summer of 2014 on the Beltsville Agricultural Research Center North Farm in Beltsville, MD. All *H*. *halys* adults and eggs used in this experiment came from our laboratory colony, which was established by Jeffrey Aldrich in 2007 with adults collected in Allentown, PA, and was reportedly supplemented annually by ~20 adults field-collected in the Beltsville area until 2010. Insects in this colony were reared in growth chambers at 25°C, 40–60% humidity and a 16L:8D cycle. They were provided with certified organic green beans (MOM’s organic market College Park, MD), hulled raw sunflower seed (Meyer Seed Co. Baltimore, MD), buckwheat seed (Meyer Seed Co. Baltimore, MD) and water *ad libitum*. Three different egg mass treatments were deployed weekly in three different habitat types.

### Sentinel egg mass treatments

*H*. *halys* masses were deployed in three different ways: fresh from rearing, frozen, and cage-laid.

#### Fresh from rearing

Fresh egg masses (≤ 24-hours-old) laid by colony insects on paper towels were collected and pinned to the underside of leaves of various vegetation using a sewing pin at each of the three sites. An additional set of fresh egg masses were collected from our colony and reared in a growth chamber at 25°C, 40–60% humidity and a 16L:8D cycle as a control treatment.

#### Frozen

Fresh egg masses (≤ 24-hours-old) laid by colony insects on paper towels were collected and immediately frozen at -80°C for 2 minutes. The egg masses were then pinned to the underside of leaves of various vegetation using a sewing pin at each of the three sites.

#### Cage-laid

Two mesh cages (30.5 cm x 30.5 cm x 61 cm with 20x20 mesh aluminum screening; BioQuip, Rancho Dominguez, CA) were secured on the ground at each site with twine and stakes where they remained for the course of the experiment. Each cage contained 15 female and 5 male *H*. *halys* adults. Adults in cages were replenished as needed and were provided with a diet of green beans, hulled sunflower seed, and buckwheat seed (as in the laboratory rearing), and one potted bush bean plant (E-Z Pick Bush Bean, Johnny’s Selected Seeds, Winslow, ME) all replaced weekly. At each site, one cage was placed in a shaded area and the other was placed in direct sunlight. Throughout the course of the experiment, the female *H*. *halys* adults laid eggs on the bush bean plants inside the cages. Parasitoids could enter though the mesh to find and parasitize the fresh cage-laid eggs.

### Habitat characteristics

To gain insight into the effect of habitat on parasitoids of *H*. *halys*, we stationed each type of egg mass in each of three contrasting habitats: soybean field, apple orchard, and second-growth woods, on the North Farm of Beltsville Agricultural Research Station, Beltsville, Maryland.

#### Soybean field

Two adjacent soybean (*Glycine max* (L.) Merr.) fields were used, the first one a 2.2 ha certified organic field (39°01’47”N 76°55’41”W) with numerous pigweed (*Amaranthus* sp.) used before 5 August 2014, and the second a 1.1 ha conventional soybean field (39°02’01”N 76°55’39”W) used thereafter.

#### Orchard

The orchard site (39°01’28”N 76°56’22”W) was an abandoned apple (*Malus domestica* Borkh.) orchard, 40m by 43m, with apple trees ~6m tall, row spacing of 5m, alleys mowed ~2x per year, with native and non-native grasses, and additional woody vegetation growing in with the apple trees, primarily Japanese honeysuckle (*Lonicera japonica* Thunb.), bush honeysuckle (*Lonicera* sp.), feral Callery pear (*Pyrus calleryana* Decne.), blackberry (*Rubus* sp.), and multiflora rose (*Rosa multiflora* Thunb. ex Murr.). To the east and north were mowed hayfields, to the south, soybeans (*Glycine max* (L.) Merr.), and to the west, a gravel road bounded by a windbreak of arborvitae (*Thuja* sp.) and bush honeysuckle (*Lonicera* sp.).

#### Woods

The woods site was an open second-growth woods adjacent (within 20m) of the west bank of the channelized Little Paint Branch (39°01’42”N 76°55’47”W). Vegetation was native and nonnative, wild, planted and invasive, with over 20 woody species within 10m, dominated by basswood (*Tilia americana* L.), American holly (*Ilex opaca* Aiton), red maple (*Acer rubrum* L.), arborvitae (*Thuja* sp.), sycamore (*Platanus occidentalis* L.), mulberry (*Morus rubra* L.), Norway maple (*Acer platanoides* L.) tree of heaven (*Ailanthus altissima* (Mill.) Swingle), black cherry (*Prunus serotina* Ehrh.), feral Callery pear (*Pyrus calleryana* Decne.), Japanese honeysuckle (*Lonicera japonica* Thunb.), bush honeysuckle (*Lonicera* sp.), grape (*Vitis* sp.), poison ivy (*Toxicodendron radicans* (L.) Kuntze), and dewberry (*Rubus hispidus* L.), with an herb layer including various grasses, mugwort (*Artemisia vulgaris* L.) and yellow rocket (*Barbarea vulgaris* R.Br.).

### Egg deployment, collection, and evaluation

We deployed each egg mass treatment on 8 dates in 2014 (July 21 and 28, August 4, 18, and 25, and September 2, 8, and 15) in each of the three habitats, all within ~20m of one another at each site. All egg masses were exposed for 72 hours in the field, at which time they were returned to the laboratory where they were held for emergence to determine survival and patterns of parasitism. Cage-laid eggs were collected from the bush bean plants after field exposure by cutting a section of leaf surrounding the egg mass. All egg masses were reared in plastic zip top bags in a growth chamber at 25°C, 40–60% humidity and a 16L:8D cycle, and checked circa daily for emergence of *H*. *halys* or parasitoids. Any parasitoids that emerged were placed in 95% ethanol for identification. Parasitoids that were either fully or partially developed, but unable to emerge from the host eggs, were extracted for identification. If this dissection was required, the egg mass was placed in a Petri dish, covered with gel hand sanitizer (62% ethanol, HDX, Atlanta, GA) to prevent live wasps from flying or the eggs from bouncing away during dissection. Dissected wasps were then placed in 95% ethanol to await identification.

### Statistical analysis

Amongst habitats and egg mass types, numbers of egg masses from which adults successfully emerged, and numbers predated, were tested as to treatment effects by Fisher’s exact test, with Freeman-Halton extension for 2xn tests where n>2 [15]. We used marginal rate analysis as described in Elkinton et. al, [16] to determine underlying parasitism rates of all sentinel eggs, including those eggs not recovered or in which we could not determine the cause of mortality.

### Parasitoid identification

Species of *Trissolcus* were identified using the key of Talamas et al. 2015 [13]; *Telenomus podisi* was identified using the key of Johnson 1984 [17]; *Anastatus reduvii* was identified using Burks 1967 [18].

## Results

Frozen egg masses yielded higher rates of successful parasitism than fresh egg masses. Of the 117 egg masses each frozen and fresh, 22 frozen egg masses, but only 7 fresh egg masses, yielded parasitoid adults, a significant difference (p = 0.0015, Fisher exact 1-tail test). On the basis of individual host eggs, 121 of a total of 2966 fresh sentinel eggs yielded adult parasitoids, 97 of which successfully emerged from the host eggs, whereas 468of a total of 2898 frozen sentinel eggs yielded adult parasitoids, 292 of which successfully emerged. Both the numbers of parasitoids maturing to adulthood, and the lesser numbers that successfully emerged from host eggs, were significantly higher in frozen eggs (χ^2^ (Pearson) = 235.3 and 101.2 respectively, p<0.0001 for both).

Four species of scelionid wasps (*Trissolcus japonicus*, *Trissolcus brochymenae*, *Trissolcus euschisti*, and *Telenomus podisi*) and one species of eupelmid (*Anastatus reduvii*) emerged from sentinel BMSB eggs, (Table 1). Seven egg masses yielded the exotic *Trissolcus japonicus* (Ashmead), a new record for North America [13]. By egg mass, native parasitoid adults were much more likely to develop from frozen egg masses, developing in 23 egg masses, 20 of which were frozen, but only 3 of which were fresh, a significant difference between egg mass types (Fisher exact test P<0.0001). However, the exotic *Tr*. *japonicus* was just as likely to emerge from fresh egg masses (4) as from frozen egg masses (2)(Fisher exact test P = 0.360, not significant). Overall, over 98% of native adult parasitoids emerging from sentinel *H*. *halys* eggs emerged from the frozen eggs, whereas over 70% of the exotic *Tr*. *japonicus* emerged from fresh eggs, reflecting its known ability to reproduce successfully on *H*. *halys* eggs in their native Asia. This difference between natives and the exotic *Tr*. *japonicus* was also highly significant (Fisher 2x2 exact test P<0.0001).

**Table 1:** Occurrence of successful egg parasitism by species, with tests for differences between fresh and frozen sentinel eggs.

	EGG MASSES					EGGS		
PARASITOID SPECIES	fresh	frozen	% frozen	P≥n for frozen		fresh	frozen	% frozen
*Anastatus reduvii*	0	3	100.0%	0.116	NS	0	39	100.0%
*Telenomus podisi*	0	4	100.0%	0.056	NS	0	16	100.0%
*Trissolcus euschisti*	2	10	83.3%	0.0139	*	4	182	97.8%
*Trissolcus brochymenae*	1	3	75.0%	0.296	NS	1	16	94.1%
*Trissolcus japonicus*	4	2	33.3%	0.360	NS	92	39	29.8%
both native *Trissolcus* spp.	3	13	81.3%	0.0205	*	5	198	97.5%
all natives	3	20	87.0%	<0.0001	***	5	253	98.1%
all species emerged	7	22	75.9%	0.0015	**	97	292	75.1%
unemerged parasitoids	9	20				127	274	
Observed rate of parasitism						7.6%	19.5%	
Marginal rate of parasitism						10.6%	23.2%	
Predation	18	22	55.0%	0.301	NS	341	408	54.5%
Undetermined mortality	45	2				509	55	
TOTAL Sentinel Egg masses / Eggs	117	117				2966	2898	

NS = not significant;

* P<0.05;

** 0.001<P<0.05;

*** P<0.001 by Fisher exact test for egg masses, using n = 117 egg masses minus number completely predated

(11 fresh and 15 frozen), or lost (2 egg masses each), as total available egg masses in which to observe parasitism frequency. Observed rate of parasitism include emerged and unemerged parasitoids; marginal rate of parasitism mb = db/(1-ma), where db = observed death rate from parasitism and ma = rate of death from predation and other causes (lost and undetermined mortality). Caged egg mass treatment is not included.

Parasitoid occurrence was very different by species among different habitats (Table 2). All three *Trissolcus* species were more likely to occur in the woods habitat (P<0.00001), whereas *Telenomus podisi* was recovered exclusively from soybeans (P≈0.00001 habitat difference), and *Anastatus reduvii* was recovered exclusively from the apple orchard (a non-significant habitat effect due to low number of egg masses parasitized). As a group, native parasitoids did not exhibit a difference in proportion of egg masses attacked by habitat, but with the inclusion of the exotic *Tr*. *japonicus*, did show a significant habitat difference (P = 0.00162), with a majority (20 of 35) of parasitized egg masses in the woods habitat.

**Table 2:** Occurrence of successful egg parasitism by species, with tests for differences among habitats, including both fresh and frozen sentinel eggs.

	EGG MASSES					EGGS		
PARASITOID SPECIES	Soy	Apple	Woods	P_A_ for difference among habitats		Soy	Apple	Woods
*Anastatus reduvii*	1	2	0	0.0658	NS	1	38	0
*Telenomus podisi*	4	0	0	0.0091	**	16	0	0
*Trissolcus euschisti*	0	2	10	< 0.0001	***	0	26	160
*Trissolcus brochymenae*	0	0	4	0.0354	*	0	0	17
*Trissolcus japonicus*	0	0	6	0.0036	**	0	0	131
both native *Trissolcus* spp.	0	2	14	< 0.0001	***	0	26	177
all natives	5	4	14	0.0295	*	17	64	177
all species emerged	5	4	20	0.0037	**	17	64	308
unemerged parasitoids	16	4	8			185	84	132
Observed rate of parasitism						10.2%	7.6%	22.8%
Marginal rate of parasitism						14.6%	9.4%	26.5%
Predation	19	11	10	0.0858	NS	360	240	149
Undetermined mortality	14	17	14			235	153	121
TOTAL Sentinel Egg masses / Eggs	78	78	78			1976	1960	1928

NS = not significant;

* P<0.05;

** 0.001<P<0.05;

*** P<0.001 by Fisher exact test for egg masses, using n = 117 egg masses minus number completely predated

Eggs laid in cages on green beans by captive *H*. *halys* females resulted in 10 of 61 egg masses laid being parasitized in soybean habitat, zero of 35 egg masses parasitized in the orchard habitat, and 2 of 57 egg masses parasitized in the woods habitat, all by *Telenomus podisi* (F.)(Hymenoptera: Scelionidae). Of these, 169 individual parasitoids developed in soy, and 29 in woods habitat, but only 29 and 2 adult wasps, respectively, emerged successfully from these eggs. Within the soy habitat, we may compare the fresh sentinel egg masses (0 of 39 parasitized) with the cage-laid egg masses (10 of 61 parasitized), showing that *Te*. *podisi* was recovered significantly more frequently from the cage-laid egg masses on common bean, than from the fresh sentinel egg masses on the surrounding soybean (Fisher 2x2 Exact test, P_1-tail_ = 0.00521). Although there is a plant species difference between common bean and soybean, these results may suggest a parasitoid preference for the caged (immediately laid) versus fresh sentinel eggs (laid over the past 24 hours and without the adult females being present in the field).

Predation did not differ by egg mass treatment (Table 1) nor by habitat type (Table 2); a mean of 17.4% of egg masses, and 13.0% of individual eggs, showed evidence of predation. Because of these eggs predated, lost, or unhatched due to undetermined causes, we used marginal rate of parasitism analysis to determine the level of parasitism in those eggs. According to marginal rate analysis (Tables 1 and 2), observed rates of parasitism ranged from 7.6% in the orchard to 22.8% in the woods habitat; the marginal rate of parasitism ranged respectively from 9.4% to 26.5%.

Of the *H*. *halys* control eggs kept in the laboratory, 81% successfully hatched (M. Cornelius, pers. comm.). It is unclear why the remaining 19% of eggs were not viable.

## Discussion

Inclusion of our wooded habitat site allowed us to be the first to detect the exotic *Trissolcus japonicus*, previously thought to only be found in quarantine in the United States [13]. Previous *H*. *halys* sentinel egg mass studies focused solely on agricultural habitats including both vegetable crops and orchards, and generally reported very low rates of parasitism. By using fresh laid eggs in screen cages we were able to detect higher levels of parasitism than fresh sentinel eggs. However, this study also suggests that *H*. *halys* egg parasitoids are habitat specific and because we only used bean plants in our screen cages, we only recovered the parasitoid wasp specific to the open soybean field habitat, *Te*. *podisi*.

Although we did not include multiple sites of each habitat type, our results are consistent with those of Okuda and Yeargan [19], who found *Telenomus* species to prefer herbaceous hosts and *Trissolcus* species to prefer woody host plants. Each parasitoid species seems to have a distinct habitat and/or host plant preference. We found only *Te*. *podisi* in the soy bean field and on the bean plants in the cage treatments, while *Trissolcus* species were found only on woody arboreal host plants at both the orchard and woods sites. *Anastatus reduvii* were found only on woody hosts at the orchard site. Further investigation into host plant and habitat preferences of these parasitoids should be conducted by replicating each habitat type.

Overall, frozen egg masses were more likely to yield adult parasitoids versus fresh. This is likely due to suppression of the immune response of the host egg due to freezing the eggs [11]. Parasitoids were also more likely to develop to adulthood in frozen egg masses versus fresh even if they were not successful at emerging from the host egg. This suggests that despite being a suitable host for completion of development to adulthood, native scelionid parasitoids are not well adapted to the invasive *H*. *halys* as a host, since they cannot exit the egg. Native parasitoids overall were less successful at both developing to adulthood and emerging from *H*. *halys* fresh and frozen egg masses than *Tr*. *japonicus* likely because the parasitoids native to North America did not co-evolve with *H*. *halys*.

Because this study relied on morphological features for identification of parasitism, we cannot exclude the possibility of multiple parasitism in egg masses in which parasitoids did not completely develop and did not emerge. Marginal rates of parasitism help to determine a more realistic level of parasitism, but molecular identification techniques would be even more helpful in illustrating an even more accurate picture of parasitism rates in the field.

It is unclear why 19% of the control eggs did not hatch in the laboratory. It is likely that a similar percentage of eggs placed in the field were also not viable. Given the low success rate of parasitism in fresh *H*. *halys* eggs (7%), this suggests the possibility that native parasitoids are only successfully parasitizing and emerging from the non-viable eggs within the fresh *H*. *halys* egg masses. Molecular techniques have the potential for determining these scelionid parasitoid- pentatomid host relationships, including where the parasitism is unsuccessful, as shown by Gariepy et al. [20].

Detection of *Tr*. *japonicus* in wooded habitat in the United States is concerning for two reasons and warrants further investigation. First, although *Tr*. *japonicus* could decrease the population of *H*. *halys* overall, this parasitoid was not found in the soybean field habitat in which *H*. *halys* can be abundant and damaging during the late summer. Second, it has been shown in the laboratory that *Tr*. *japonicus* readily and successfully parasitizes eggs of native beneficial stink bugs including the spined soldier bug, *Podisus maculiventris* (Say), which is an important predator of both native and invasive pests such as Mexican bean beetle (*Epilachna varivestis* Mulsant (Coleoptera: Coccinellidae), Colorado potato beetle (*Leptinotarsa decemlineata* (Say)(Coleoptera: Chrysomelidae)), cabbage looper (*Trichoplusia ni* (Hübner)(Lepidoptera: Noctuidae), and gypsy moth (*Lymantria dispar* (L.)(Lepidoptera: Lymantriidae)) [10–24]. Given that woody habitats are preferred for overwintering and in the early season by *P*. *maculiventris* [25], and that our wooded site is the only one where *Tr*. *japonicus* was detected, this poses non-target concerns and will be explored in more detail in future field studies.

## Supporting Information

S1 Data All raw data is contained in the supporting information file, “Herlihy 2014 data.xlsx.” (XLSX)

## References

[1] Hoebeke ER, Carter ME. Halyomorpha halys (Stål) (Heteroptera: Pentatomidae): A polyphagous plant pest from Asia newly detected in North America. Proc. of the Entomol Soc of Washington 2003; 105:225–237.

[2] Haye T, Gariepy TD, Heolmer K, Rossi JP, Streito JC, Tassus X, et al expansion of the invasive brown marmorated stinkbug, Halyomorpha halys: an increasing threat to field, fruit and vegetable crops worldwide. J of Pest Sci.2015 10.1007/s10340-015-0670-2

[3] Leskey TC, Hamilton GC, Nielsen AL, Polk DF, Rodriguez-Saona C, Berg JC, et al Pest status of the brown marmorated stink bug, Halyomorpha halys (Stål), in the USA. Outlooks on Pest Management 2012; 23: 218–226.

[4] Northeastern IPM Center. Where is BMSB? 2014. Available: http://www.stopbmsb.org/where-is-bmsb/host-plants/. Accessed October 2015.

[5] Northeastern IPM Center. Host Plants of the Brown Marmorated Stink Bug in the U.S. 2014. Available: http://www.stopbmsb.org/where-is-bmsb/host-plants/. Accessed October 2015.

[6] Rice K, Bergh C, Bergman E, Biddinger D, Dieckhoff C, Dively G, et al Biology, Ecology, and Management of Brown Marmorated Stink Bug (Halyomorpha halys) J of Integ Pest Management 2014; 5(3): A1–A13. Available: 10.1603/IPM14002.

[7] Weber DC, Leskey TC, Walsh GC, Khrimian A. Synergy of aggregation pheromone with methyl (E, E, Z) -2, 4, 6-decatrienoate in attraction of Halyomorpha halys (Hemiptera: Pentatomidae). J of Econ Entomol 2014; 107(3):1061–8. 25026665 10.1603/ec13502

[8] Leskey TC, Agnello JA, Bergh C, Dively GP, Hamilton GP, Jentsch P, et al Attraction of the invasive Halyomorpha halys (Hemiptera: Pentatomidae) to traps baited with semiochemical stimuli across the United States. Environ Entomol 2015; 44:746–756. 10.1093/ee/nvv049 26313981

[9] Jones AL, Jennings DE, Hooks CRR, Shrewsbury PM. Sentinel eggs underestimates rates of parasitism of the exotic brown marmorated stink bug, *Halyomorpha halys*. Biol Control 2014; 78: 61–66.

[10] Abram PK, Gariepy TD, Boivin G, Brodeur J. An invasive stink bug as an evolutionary trap for an indigenous egg parasitoid. Biol Invasions 2014; 16(7), 1387–1395.

[11] Haye T, Fischer S, Zhang J, Gariepy T. Can native egg parasitoids adopt the invasive brown marmorated stink bug, Halyomorpha halys (Heteroptera: Pentatomidae), in Europe? J of Pest Sci 2015 10.1007/s10340-015-0671-1

[12] Talamas EJ, Johnson NF, Buffington ML. Key to the Nearctic species of Trissolcus Ashmead, natural enemies of native and invasive stink bugs (Pentatomidae). J of Hymen Res 2015; 43: 45–100.

[13] Talamas EJ, Herlihy MV, Dieckhoff C, Hoelmer K, Buffington M, Bon MC, et al Trissolcus japonicus (Ashmead) (Hymenoptera, Scelionidae) emerges in North America. J of Hymen Res 2015; 43: 119–128.

[14] Vet LEM, Dicke M. Ecology of Infochemical use by natural enemies in a tritrophic context. Annual Review of Entomology 1992; 37: 141–172.

[15] Lowry, R. VassarStats:Website for Statistical Computation. 2015. Available: http://vassarstats.net. Accessed August 2014.

[16] Elkinton JS, Buonaccorsi JP, Bellows TS, Van Driesche RG. Marginal attack rate, k-values and density dependence in the analysis of contemporaneous mortality factors. Res on Pop Ecol 1992; 34:29–44.

[17] Johnson NF. Systematics of Nearctic Telenomus: classification and revisions of the podisi and phymatae species groups (Hymenoptera: Scelionidae). Bulletin of the Ohio Biological Survey 1984; 6: 1–113.

[18] Burks BD. The North American species of Anastatus Motschulsky (Hymenoptera, Eupelmidae). Trans American Entomological Society 1967; 93: 423–432.

[19] Okuda MS, Yeargan KV. Habitat partitioning by Telenomus podisi and Trissolcus euschisti (Hymenoptera: Scelionidae) between herbaceous and woody host plants. Environ Entomol 1998; 17: 795–798.

[20] Gariepy TD, Haye T, Zhang J. A molecular diagnostic tool for the preliminary assessment of host—parasitoid associations in biological control programmes for a new invasive pest. Molec Ecol 2014; 23: 3912–3924. 24102670 10.1111/mec.12515

[21] McPherson JE. A list of the prey species of Podisus maculiventris (Hemiptera: Pentatomidae). Great Lakes Entomol. 1980; 13: 17–24.

[22] Hoffmann MP, Frodsham AC. Natural Enemies of Vegetable Insect Pests. Cornell University, Ithaca, NY, 1993, 63 pp.

[23] Wiedenmann RN, Legaspi JC, O'Neil RJ. Impact of prey density and facultative plant feeding on the life history of the predator Podisus maculiventris (Say), pp. 94–118. In: Alomar O. and Wiedenmann R. N., eds. Zoo-phytophagous Heteroptera: Implications for life history and IPM. Thomas Say Publications in Entomology, 1996.

[24] Montemayor CO, Cave RD. Development time and predation rate of Podisus maculiventris (Hemiptera: Pentatomidae) feeding on Microtheca ochroloma (Coleoptera: Chrysomelidae). Environ Entomol 2011; 40: 948–954. 10.1603/EN10275 22251696

[25] Jones WA, Sullivan MJ. Overwintering habitats, spring emergence patterns, and winter mortality of some South Carolina Hemiptera. Environ Entomol 1981; 10: 409–414.

